# A developmental framework of interpersonal neural synchrony

**DOI:** 10.1016/j.dr.2025.101234

**Published:** 2025-12

**Authors:** Stefanie Hoehl, Anna Bánki, Alicja Brzozowska, Alessandro Carollo, Kathrin Kostorz, Trinh Nguyen, Carolina Pletti, Susanne Reisner, Verena T. Schäfer, Christina Schaetz, Markus R. Tünte

**Affiliations:** a Faculty of Psychology, University of Vienna, Vienna, Austria; b Faculty of Rehabilitation Sciences, Technical University of Dortmund, Dortmund, Germany; c Department of Psychology and Cognitive Science, University of Trento, 38068 Rovereto, Italy; d Neuroscience of Perception and Action Lab, Italian Institute of Technology, Rome, Italy; e Department of Developmental and Biological Psychology, Heidelberg University, Heidelberg, Germany; f Vienna Doctoral School Cognition, Behavior and Neuroscience, University of Vienna, Vienna, Austria

## Abstract

Interpersonal neural synchrony (INS), the temporal alignment of brain activities between individuals, has been proposed as a biomarker for successful communication and smooth social interaction. Surging empirical evidence shows that INS emerges spontaneously between infants, children, and their caregivers from early on in development. Yet, little is known about the developmental preconditions and functions of INS in childhood. This paper presents a developmental framework for understanding INS, integrating insights from structural and functional brain maturation, as well as behavioral, social, and cognitive development. We discuss how early caregiver-infant interactions, characterized by shared perceptual rhythms, facilitate the emergence of INS. Given initial limitations in temporal precision of neural processing, early INS is likely constrained to low-frequency brain rhythms and evolves alongside the maturation of neural networks and socio-cognitive abilities. We outline how INS may support critical developmental processes, including social learning, language acquisition, and attachment formation, through enabling mutual prediction and co-regulation between caregivers and children. Furthermore, we hypothesize that tasks requiring higher-order mutual understanding are linked to qualitative changes in INS patterns over time. This framework highlights the potential of INS as both a marker and a driver of developmental change, offering new avenues for research and intervention. Longitudinal studies and rhythm-based interventions could deepen our understanding of how INS supports development, with implications for enhancing social learning and attachment in populations at risk for developmental challenges. This work underscores the importance of adopting a developmental perspective in INS research.

## Introduction

From the very first moments of life, infants engage with their caregivers through vocalizations, eye contact, and sharing of affect. These early social interactions shape cognitive and emotional development, serving as a framework for acquiring communicative and self-regulatory skills and laying the foundations for social bonding (Feldman, 2017; Leclere et al., 2014; Murray & Trevarthen, 1985; Stern, 1985). Beyond observable behaviors, a growing body of research in social and relational neuroscience has demonstrated that both early and adult social interactions entail interpersonal dynamics at the neural and physiological level (De Felice et al., 2025). When two people, such as a caregiver and their child, engage in social interactions, their behaviors, as well as their physiological and neural activity, tend to align. The phenomenon of mutually aligning behavioral, physiological, and brain rhythms between interaction partners is known as biobehavioral synchrony (Carollo et al., 2021; Feldman, 2012a, 2012b, 2017).

Advances in brain imaging, particularly the introduction of hyperscanning (Montague, 2002), have enabled researchers to investigate interpersonal neural synchrony (INS), i.e., the temporal alignment of neural activity between individuals, from infancy to adulthood (Carollo & Esposito, 2024). INS between adults has been consistently related to positive interactional outcomes such as successful communication (Dumas et al., 2010; Hasson et al., 2012) and cooperation (Cui et al., 2012; Czeszumski et al., 2022; Lotter et al., 2023). Emerging theoretical frameworks have linked INS to central interpersonal concepts such as mutual prediction (Hamilton, 2021; Koban et al., 2019), social learning (Shamay-Tsoory, 2021), co-regulation, and social reward (Gvirts & Perlmutter, 2020; Hoehl et al., 2021). However, these frameworks have so far neglected the specific role that INS may play in human development and, conversely, the role that socio-cognitive, motor, and brain development play for INS.

In caregiver-child dyads, INS has been observed across various experimental tasks, from co-watching a video to cooperative problem-solving and across different developmental stages (Alonso et al., 2024; Azhari et al., 2019; Nguyen et al., 2020b, 2023; Piazza et al., 2020; Reindl et al., 2018; Roche et al., 2025). Despite growing scientific interest in INS in the context of development, a theoretical developmental framework is currently lacking. At the current stage, this gap undermines researchers’ ability to design robust, theory-driven experimental tasks and formulate specific, developmentally informed hypotheses for INS studies. Yet, research in this field must embrace a developmental perspective, incorporating theoretical considerations specific to the study of the developing brain. On the one hand, future research should consider the uniquely developmental functions of INS as a potential mechanism driving the refinement of socio-cognitive and emotional processes, including communication, co-regulation, and bonding (Hoehl et al., 2021; Markova et al., 2019; Mayo & Shamay-Tsoory, 2024; Roche et al., 2025). On the other hand, it is essential to consider how the processes necessary for establishing INS may differ when they occur in brains still undergoing rapid maturation, as compared to adult brains. Early brain maturation entails increasing functional specialization of social brain networks over childhood (Johnson, 2007; Richardson et al., 2018) and a steep acceleration of information processing from infancy to adulthood (Hochmann & Kouider, 2022b, 2022a), both of which likely constrain the precision of social coordination processes in young children (Su et al., 2020).

Our paper aims to provide a developmental framework of INS by integrating insights from research on structural and functional brain maturation, as well as behavioral, social, and cognitive development. We seek to provide an integrative framework that can guide the interpretation of INS research findings in developmental populations and inspire the formulation of new developmentally informed hypotheses. We start by outlining how the (developing) brain synchronizes with external perceptual signals, such as auditory and visual rhythms, a process that is also referred to as entrainment. The synchronization of brain rhythms with perceptual rhythms is a basic process, not restricted to socially exchanged signals per se, yet of great importance for social interactions. Mutually entraining brain rhythms to each other’s communicative signals during interactions is considered one of the key mechanisms underlying INS (Hasson et al., 2012). Hence, we discuss how rhythms are processed in the developing brain as a basic prerequisite for INS. In addition to external perceptual rhythms, we also examine the processing of internal rhythms, such as heartbeat—an integral component of interoception, i.e., the perception of bodily signals. This internal rhythm processing has important implications for biobehavioral synchrony. Next, we discuss the production of synchronized motor output, which requires coordinated motor control alongside precise rhythm perception. In the second half of the paper, we then move on from basic perceptual, attentional, and sensorimotor mechanisms to more complex processes of synchronizing in social interactions, including higher-order conceptual alignment. We critically discuss the potential role of INS as a developmental mechanism and point to important future directions for research and application.

## Synchronizing with perceptual rhythms

Perceptual rhythms, such as speech, play an important role in establishing INS between interaction partners (De Felice et al., 2025; Hasson et al., 2012; Hoehl et al., 2021; Schilbach & Redcay, 2025). In reciprocal face-to-face interactions, partners synchronize their oscillatory brain activities with the rhythms of each other’s communicative signals, thus facilitating communication and establishing INS (Hasson et al., 2012; Hasson & Frith, 2016; Wass et al., 2020). This pathway to INS may be especially relevant in early development as caregivers intuitively provide infants with rich rhythmic stimulation (Markova et al., 2019). A prominent proposal is that infants and caregivers synchronize their neural activities through shared perceptual and communicative rhythms in their social exchanges, such as infant-directed speech (Nguyen et al., 2023; Piazza et al., 2020) and singing (Lense et al., 2022; Nguyen et al., 2023; Markova et al., 2020). Yet, a fundamental prerequisite for this proposition is that the infant brain is capable of synchronizing neural activity to communicative rhythms in a similar way as the adult brain (Hasson et al., 2012). Given the prolonged anatomical and functional maturation of the human brain, this question warrants careful consideration. In our developmental framework of INS, we thus first turn our attention to the infant brain’s ability to synchronize with external and internal perceptual rhythms.

### Neural mechanisms of synchronizing with perceptual rhythms

Synchronizing with external perceptual rhythms in social interactions is thought to rely on the phenomenon of neural entrainment – the alignment of neural oscillations with an external periodic force, such as rhythmically presented auditory or visual stimuli (Buzsáki, 2006; Obleser & Kayser, 2019; Thut et al., 2011; Zoefel et al., 2018). It is still a matter of ongoing debate whether endogenous brain rhythms are modulated by this process, reflecting so-called neural entrainment in the narrow sense, or whether rapid event-related brain responses track rhythmic stimuli, sometimes referred to as neural entrainment in the broader sense (Bánki et al., 2022; Doelling & Assaneo, 2021; Keitel et al., 2014). In any case, the temporal alignment of neural activity with external stimuli supports sensory processing, acting as an amplifier for rhythmic input (Buzsáki & Draguhn, 2004; Calderone et al., 2014; Schroeder & Lakatos, 2009). Therefore, entrainment is considered a core mechanism for facilitating perceptual and cognitive processes such as selective attention (Calderone et al., 2014), vocal speech perception (Zion Golumbic et al., 2013), and social interactions (Markova et al., 2019; Wass et al., 2020).

Infants and children encounter rhythmic information across multiple sensory modalities. Most developmental research focuses on visual and auditory domains, such as when infants observe flickering images (Köster et al., 2023), listen to music (Nguyen et al., 2023), or speech (Menn et al., 2022; Ortiz Barajas et al., 2021). While these modalities are often studied separately, infants typically experience them together and appear to integrate both to support entrainment (Ní Choisdealbha et al., 2024). Artificial rhythms, such as metronomic beats or flickering stimuli, are often used in controlled experimental research as they can be applied with high temporal precision and periodicity (Kabdebon et al., 2022; Köster et al., 2023). Meanwhile, naturalistic rhythms in movement, speech, or interactive music involve greater variability, allowing researchers to tap into more naturalistic scenarios of early social communication (Menn et al., 2022; Nguyen, Reisner, et al., 2023; Nguyen, Zimmer, et al., 2023; Phillips et al., 2024).

Due to the high temporal resolution required to monitor these processes, neural entrainment to sensory rhythms is typically captured with electroencephalography (EEG) or, though less common in developmental research, with magnetoencephalography (MEG) (Schwab et al., 2006; Tal et al., 2017). The quantification of neural entrainment can be broadly categorized into three approaches: those focusing on amplitude or power, those focusing on phase information, and those focusing on both (see Kabdebon et al., 2022 for methodological recommendations for implementing developmental studies on neural entrainment). The first approach takes advantage of the fact that a rhythmic perceptual signal at a given frequency causes a narrowband peak at that same frequency to appear in the EEG amplitude or power spectrum due to the entrainment. Comparing the amplitude/power at that peak with the amplitude/power at neighboring frequencies (or frequency bins) yields a signal-to-noise ratio index, which captures the strength of neural processing of the rhythmic stimulus of interest. As in adults, signal to noise ratios are modulated by overt and covert attention in infants (Bánki et al., 2024; Christodoulou et al., 2018; Robertson et al., 2012) and are easily comparable across individuals and groups (Cohen & Gulbinaite, 2017; Peykarjou et al., 2024). The alternative, complementary approach to measuring neural entrainment focuses on the phase information. It quantifies the exact temporal alignment of the neural response with the perceptual rhythm. An entrained neural response that exhibits a steady phase relation to the perceptual rhythm would yield high phase coherence across stimulation cycles, indicating high temporal alignment (Duecker et al., 2024). Additionally, it is interesting to examine the preferred phase of this alignment, that is the specific angle around which the phase responses concentrate. For instance, research on language development demonstrated an association between the preferred phase and language performance in the first two years of life (Ní Choisdealbha et al., 2023) as well as the occurrence of developmental dyslexia in later childhood (Colling et al., 2017; Power et al., 2013), suggesting the added value of incorporating phase information into neural entrainment research.

Finally, some measures of neural entrainment in early development incorporate amplitude and phase information at the same time (Attaheri et al., 2022; Menn et al., 2022; Nguyen et al., 2023; Bianco et al., 2025). For instance, recent research on neural entrainment to speech rhythms used coherence, which relates the infant’s neural signal to the speech signal in terms of their phase-synchronization, weighted by their relative amplitude (Menn et al., 2022). Neural tracking of naturalistic external rhythms with variability can be quantified this way and responses in specific frequency bands of interest can be separated. Analytical approaches that relate features of the brain response to the features of dynamic, multimodal perceptual input streams (e.g., multivariate temporal response function/mTRF) allow for quantifying neural entrainment in naturalistic contexts, and are increasingly applied to developmental populations (Jessen et al., 2021).

### Temporal precision across early development

The processing of temporal regularities in sensory input streams is constrained by brain maturation as the temporal precision of sensory perception undergoes development until several years of age. In the visual domain, sensitivity to temporal changes in 6-month-olds is adult-like for low-level features such as luminance (Apkarian, 1993; Saint et al., 2017), but continues to develop until around 5 years of age for more complex features (Freschl et al., 2019). The gradual improvements in the temporal resolution of visual attention have been linked to changes in oscillatory neural activity throughout development, particularly in individual peak frequency of the alpha rhythm (Arioli et al., 2024; Freschl et al., 2022), which spans roughly between 4 and 10 Hz in the infant EEG and between 8 and 12 Hz in the adult EEG (Saby & Marshall, 2012). The higher the frequency at which the alpha rhythm exhibits maximum amplitude, the better the temporal resolution of visual attention – both in adults (Samaha & Postle, 2015) and in infants (Arioli et al., 2024). This developmental shift from slower to faster rhythms (and the accompanying progress in visual temporal perception) is thought to reflect increasing axonal myelination (Caffarra et al., 2024; Valdés-Hernández et al., 2010) and gradual formation of corticothalamic connections (Clayton et al., 2018; Minami et al., 2020). While relatively less researched, in the auditory domain, the temporal resolution of perception continues to develop until late childhood (Balen et al., 2009; Litovsky, 2015). Finally, multisensory temporal perception develops rapidly in the first year of life and continues to improve as a function of neural maturation and perceptual experience (Lewkowicz, 2012).

These developmental changes in the temporal precision of perception likely affect INS through fine-tuning temporal interpersonal coordination, especially in interactions that involve turn-taking (Nguyen et al., 2023; Templeton et al., 2022). Less precise temporal processing may entail a relatively higher tolerance to stimulus jitter in the infant compared to the adult brain, meaning that the infant brain may entrain to less strictly periodic stimuli. Indeed, there is initial evidence that 4–6-month-olds’ brains entrain to an 8 Hz auditory rhythm with jittered beats, whereas adults show decreasing entrainment with increasing jitter levels (White, 2023). Notably, initial deficits in processing speed in the human brain may provide infants with functional perceptual bottlenecks (Vogelsang et al., 2024). By presenting a low-pass filter for sensory input, initial limitations in temporal resolution may cause the developing brain to focus on low-frequency information. This could facilitate the formation of long-range neural connections and perceptual integration, as needed, for instance, when processing low-frequency auditory information such as prosody in speech (Vogelsang et al., 2024). Research targeting these processes in infancy and childhood thus benefits from careful consideration of the timescales at which the relevant signals operate (Piazza et al., 2021; Hoehl and Bertenthal, 2021), and how their perception may differ across development.

### Maturation of brain structures involved in synchronizing with perceptual rhythms

Synchronizing with external perceptual rhythms is subserved by timing- and modality-specific brain structures that seem to come online remarkably early in life. Despite the initially limited temporal precision of early visual processing, there is evidence for visual cortical entrainment to simple gratings presented at 10 Hz in human neonates (Atkinson et al., 1979). In the auditory domain, pre-maturely born infants at 32 weeks of gestational age were shown to entrain EEG activity to a 3 Hz beat (Edalati et al., 2023). These results indicate early maturation of brain structures involved in passively tracking environmental rhythms and their effective connectivity by the third trimester of gestation. Neuroimaging and lesion studies in adults point to the striatum as a central timer in the brain (Coull et al., 2011; Merchant et al., 2013). According to the *striatal beat frequency model*, deriving temporal regularities from sensory input relies on striato-cortical loops and dopaminergic signaling (Buhusi & Meck, 2005). Developmental brain imaging research has revealed rapid structural maturation of the striatum in the second trimester (Tian et al., 2022) and functional connectivity between the striatum and sensorimotor areas in early childhood (Choi et al., 2023).

While the human brain passively represents temporal regularities in sensory input from birth, and likely even prenatally, selective dynamic attention allows us to focus on relevant incoming sensory streams and to disregard others (Henry & Herrmann, 2014; Jones, 1976; Jones et al., 2002). For instance, adults’ EEG amplitudes at the flicker frequency are increased when a flickering visual stimulus is covertly spatially attended (Muller et al., 1998). This selective dynamic attention likely relies on fronto-striatal networks subserving cognitive control and attention, which display protracted development compared to sensorimotor-striatal connections (Choi et al., 2023; Durston & Casey, 2006). Remarkably, attentional modulations of infant flicker-frequency specific EEG amplitudes have been documented by 3–4 months of age (Christodoulou et al., 2018; Robertson et al., 2012). This indicates an early emerging ability of the infant brain to tune to specific rhythms. Indeed, attention control – the ability to select what is attended to or filtered out – develops early in infancy (Amso & Scerif, 2015; Oakes, 2023). Neonates fixate on schematic faces longer than on other stimuli (Johnson et al., 1991), which seems to be reflected in enhanced EEG responses to slowly flickering face-like stimuli (Buiatti et al., 2019). By 3–6 months, infants acquire voluntary control of visual fixation and scanning, allowing them to focus on relevant stimuli for further processing and learning (Braddick & Atkinson, 2011; Reynolds, 2015). By this age, infants gradually start to develop endogenous attention control (Krieber-Tomantschger et al., 2022). The development of attention control is shaped by infants’ engagement in social interactions with their caregivers (Ilyka et al., 2021; Wass et al., 2022, 2024; Hoehl and Bertenthal, 2021), and in particular, by caregivers’ responsiveness and predictability (Lancaster & Wass, 2024).

Whereas adults selectively entrain to rhythms with a high social and communicative value (Hoehl et al., 2021), it is less clear when this preference for complex social rhythms emerges in human ontogeny. In addition to the described striato-cortical loops, superior temporal brain regions are thought to play an important role in this process by linking temporal processing to the social brain (Hoehl et al., 2021). The temporo-parietal junction (TPJ) and superior temporal sulcus (STS) are considered key areas in the social brain network, and are involved in integrating multimodal cues (Patel et al., 2019), social attention (Ahmad et al., 2021), and social perception (Lee Masson et al., 2024; Lee Masson & Isik, 2021; Pelphrey et al., 2003). The TPJ and STS integrate bottom-up sensory inputs with top-down cognitive processes to support social perception, perspective taking, and interaction monitoring (Carter & Huettel, 2013; Doricchi et al., 2023; Patel et al., 2019). The right TPJ, in particular, has been consistently implicated in INS across a wide variety of studies and tasks (Lotter et al., 2023).

Structural and functional maturation shape the role of these structures in social cognition over time (Mills et al., 2014; Schüler et al., 2024; Van Der Meulen et al., 2023). The TPJ undergoes significant structural changes in early life, with steep increases in myelination documented between 2 and 36 months (Schneider et al., 2022). This rapid maturation may lay the foundation for higher-order social processing, such as mental perspective taking, which continues to evolve through increasing connectivity with other regions. A key region interacting with the TPJ in social cognition is the posterior STS, which plays a central role in detecting social cues such as facial expressions, gaze, and motion (Haxby et al., 2000; Patel et al., 2019). As development progresses, increasing connectivity between posterior STS and higher-order networks, including the TPJ, supports the transition from basic social perception to complex social inference (Schüler et al., 2024). Selective neural entrainment to complex and socially relevant rhythms may thus be refined across the first years. This developmental hypothesis has yet to be tested empirically.

The maturation of social brain regions extends beyond structural development and is actively shaped by experience-dependent plasticity. Very early in development, neonatal brainstem input presents a key gateway for the bottom-up regulation of social attention, with consequences of early brainstem dysfunction on social orienting in later childhood (Geva et al., 2017). Caregiver-infant interactions contribute to the development of functional connectivity networks involved in emotion regulation and social cognition (Ilyka et al., 2021; Ulmer Yaniv et al., 2021). For instance, higher attachment security at 15 months has been associated with increased gray matter volume in key social brain regions, including the TPJ and STS, when measured at 10–11 years of age (Leblanc et al., 2017). The described developmental progression underscores the socially modulated, dynamic nature of TPJ and STS maturation, providing the neural foundation for increasingly complex social processing and interaction from infancy through adulthood. Consequently, future research should trace the development of selective neural entrainment for complex social rhythms and link individual differences to children’s early experiences in their social relationships.

### Sensitivity to bodily rhythms involved in social interactions

In addition to external perceptual rhythms, including visual and auditory communicative signals, internal bodily rhythms are closely linked with rhythmic brain oscillations (Criscuolo et al., 2022; Klimesch, 2013) and may play an integral role in the emergence of INS. For instance, social touch has been linked to INS both in adult romantic partners (Goldstein et al., 2018) and in caregiver-infant dyads (Nguyen et al., 2021), likely mediated at least in part by the transmission of rhythmic physiological signals such as breathing and heart rate in close proximity (Van Puyvelde et al., 2015; Waters et al., 2017). Thus, both the ability to track one’s own and others’ bodily rhythms may affect INS.

The perception of one’s own bodily signals is termed interoception, and has been related to affective experience, mental health, and self-perception (Khalsa et al., 2018). Many of these signals are rhythmic. In adults, a heartbeat occurs approximately once every second, while one breath takes around four seconds, and brain-body coupling can be observed for these modalities at similar frequencies (Fleming et al., 2011). One operationalization of physiological synchrony commonly used in developmental studies is respiratory sinus arrhythmia, which is an index of the coupling between heartbeat and respiration (Garcia et al., 2013; Miller et al., 2023). The stomach is also coupled to the brain, though communication is slower, with an approximate period of 20 s (Rebollo et al., 2021). Heartbeat, stomach, and respiration are integrated on a neural level, with recent approaches outlining neurocognitive models to describe their impact (Brændholt et al., 2023; Candia-Rivera et al., 2024; Rebollo et al., 2021). Although relatively less researched, neural responses to bodily signals are similar to the ones described for other perceptual rhythms in the previous sections. Evoked responses to own heartbeat or respiration can be measured via EEG in adults (Coll et al., 2021; Davenport et al., 2000) and infants (Maister et al., 2017). A recent study with adults further showed that visual EEG responses to flickering stimuli are modulated when stimuli are presented together with the adult participants’ heartbeat (Ren et al., 2024), pointing again to the close link between bodily and brain rhythms and the role of multimodal integration in rhythm processing.

Most research on the perception of bodily signals has been conducted in adults. However, there is a growing literature on the perception of bodily signals throughout infancy and childhood. Infancy might present a particularly relevant period, as bodily rhythms such as heartbeat and respiration slow down in the first two years of life (Fleming et al., 2011). In addition, infants depend on their caregivers to regulate their own bodily signals: in the case of hunger or distress, infants must communicate their needs to the primary caregiver, who in turn needs to show an appropriate reaction, highlighting the relevance of bodily signals in early interaction (Atzil et al., 2018; Fotopoulou & Tsakiris, 2017). Infants can already perceive their own cardiac and respiratory signals, as evidenced by their ability to match these signals to synchronous visual rhythms by 3 months of age (Maister et al., 2017; Tünte et al., 2025). Further, cardiac perception in 6-month-old infants is positively related to mutual gaze durations between infant and caregiver, highlighting its involvement in social interactions (Imafuku et al., 2023). Still, research on the perception of bodily signals and their coupling with the brain early in life is sparse, and more work using a variety of methodological or longitudinal approaches is needed to better understand the intra- and interpersonal dynamics of physiological and brain rhythms involved in INS.

### From neural entrainment to synchronized action

So far, we have focused on neural entrainment to external and internal perceptual rhythms as a prerequisite for establishing INS in social interactions. A further important aspect to consider is whether neural entrainment to sensory rhythms leads to synchronized motor output that can be used to coordinate and synchronize with other people (Dumas & Fairhurst, 2021; Sebanz et al., 2006), for instance, during dance (Keller et al., 2014). In the adult brain, sensory and motor areas collaborate in processing rhythms, reflecting a tightly linked perception–action loop (Grahn & Brett, 2007). Such connections emerge early (Provasi et al., 2014). First, infants passively experience synchronized actions through others – from being rhythmically moved while being carried (Rocha et al., 2021b) to being bounced by a caregiver (Cirelli et al., 2018; Phillips-Silver & Trainor, 2005). Already in the womb, a salient source of rhythmic stimulation are the mother’s footsteps, (Rocha et al., 2021b; Trevarthen, 1999), which entail acoustic, proprioceptive and vestibular stimulation for the fetus (Provasi et al., 2021), and can impact fetal cardiac activity (Lecanuet & Jacquet, 2002; Lecanuet & Schaal, 2002). Moreover, already in the womb the mother’s heartbeat is detectable and dynamic patterns of maternal-fetus cardiac coupling might already be present (Candia-Rivera & Chavez, 2025).

Next to being moved by and with others, infants also move by themselves. They spontaneously move to music even before birth and continue to do so throughout infancy (Kisilevsky et al., 2004; Nguyen et al., 2025; Zentner & Eerola, 2010). While their movement is not yet perfectly synchronized with external rhythms, infants can adjust their movement speed to tempo changes (Rocha et al., 2021a; Rocha & Addyman, 2022). By preschool age, children can synchronize their motor behavior to various metronome beats (Provasi & Bobin-Bègue, 2003). Beyond auditory input, infants’ and children’s movement is shaped by social and visual cues: By 12 months of age, infants prefer when others move synchronously with them (Tuncgenc et al., 2015). Toddlers move more synchronously when engaged in joint drumming with a human partner compared to a drumming machine or a robot (Kirschner & Tomasello, 2009; Yu & Myowa, 2021), providing evidence for preferential entrainment to social rhythms in early childhood. By age 4–6, children exhibit clearer motor synchronization with others, and movement synchrony even facilitates prosocial peer interactions (Tuncgenc & Cohen, 2018). But how do infants begin to transform sensory rhythms into synchronized actions?

Postural and locomotor control are the basis of such synchronized motor output. Though motor development varies depending on individual and cultural factors (Adolph et al., 2011; Adolph & Franchak, 2017), infants generally master bodily control in the direction from the head downwards to their shoulders, waist, and hips (Hopkins & Rönnqvist, 2002; Saavedra et al., 2012). Though progress may not be linear, infants generally support their head and upper body with their arms on the floor or furniture before learning to sit without arm support, and they subsequently learn to stabilize themselves on two feet with the support of furniture before standing freely (Atun-Einy et al., 2012; WHO Multicentre Growth Reference Study Group & De Onis, 2006). Unsupported sitting and standing allow infants to move their arms freely and twist their torso and head (for a review, see Adolph & Franchak, 2017). While limb movements are already present from the eighth gestational week (De Vries et al., 1982), they are still rather uncoordinated in early development. Leg movements go through a phase of coupling of the hip, knee, and ankle joints until 4 months of age, decoupling at 5 months, and the re-emergence of joint coupling by 8 months of age (Thelen, 1985). As infants mature, left and right limbs become more coordinated with each other, as do ipsilateral arms and legs, while joint movements within the same limb become more independent (Piek et al., 2002; Piek & Gasson, 1999). Coordinating the arms and legs is necessary for skills like walking, leg kicking, and clapping. Rhythmic arm movements peak in co-occurrence with canonical babbling (Ejiri, 1998), and infants start clapping by 8 to 12 months (Kaye & Marcus, 1981), showing the ability of sensory-motor coordination.

**Fig. 1:**
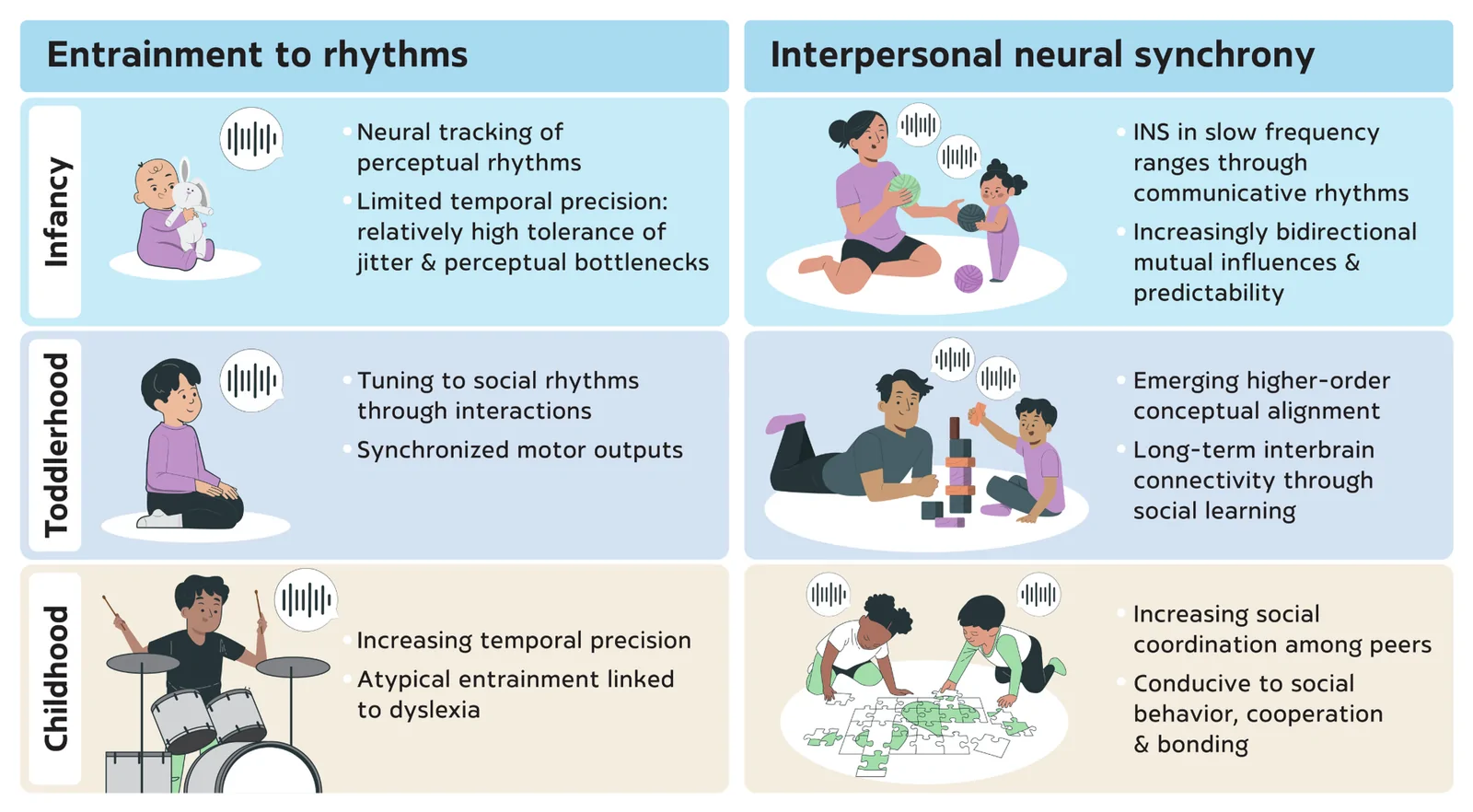
Summary of the main developmental changes in entrainment to rhythms and INS across childhood. Image by Adi Ramot.

These early developments in motor control and spontaneous rhythmic movement reflect a growing capacity for infants to react to external rhythms and actively participate in rhythmic social interactions. As their sensorimotor systems mature, infants become increasingly capable of aligning their actions with the rhythmic patterns of others. Importantly, these synchronous movements are not only products of neural entrainment but are also socially embedded experiences (see Hoehl et al., 2021). Being moved with or in response to others, and eventually moving in synchrony with them, forms the foundation of later interpersonal coordination. Studies have shown that such synchronous experiences can promote prosocial behavior (Cirelli et al., 2014; Tuncgenc & Cohen, 2018), suggesting that early motor synchrony plays multiple roles, such as facilitating sensorimotor development and social connectedness. Fig. 1 summarizes the main developmental changes in entrainment to rhythms and INS across childhood.

## Synchrony in social exchanges

Synchronizing brain and motor rhythms with external and internal perceptual rhythms provides the foundation for interpersonal synchrony, including INS (Hasson et al., 2012). INS is typically conceptualized as a dynamic and bidirectional process, requiring mutual engagement (De Felice et al., 2025). Fluctuations in the attention of interaction partners and dynamic changes in mutual attentional alignment affect INS over the course of a social exchange (Dikker et al., 2017; Mayo & Gordon, 2020). Research into early caregiver-infant interactions shows that even very young infants play an active role in this process (Beebe et al., 2016; Feldman, 2012b; Hoehl & Bertenthal, 2021). Rather than reflecting unidirectional adaptation, early social interactions are thus characterized by a reciprocal exchange of information (Cohn & Tronick, 1988). Measuring these bidirectional processes of mutual adaptation in real-time on the neural level requires hyperscanning, i.e., the recording of brain data from multiple interacting persons simultaneously. In the following, we outline empirical findings on INS in early development and discuss developmental processes and potential functions of INS from an interactionist perspective (Hoehl & Bertenthal, 2021).

### Interpersonal neural synchrony and mutual prediction across early development

In recent years, INS has been increasingly studied in real-time, reciprocal social exchanges, including early social interactions between infants and their caregivers (Alonso et al., 2024; De Felice et al., 2025; Markova et al., 2019; Nguyen et al., 2020; Roche et al., 2025; Turk et al., 2022; Wass et al., 2020; Wass & Goupil, 2022). Studies employing EEG hyperscanning have applied measures of coherence (Leong et al., 2017), power correlations (Marriott Haresign et al., 2022), phase-locking value (Kayhan et al., 2022), or weighted phase lag index (Endevelt-Shapira et al., 2021) to quantify INS between two (or more) brain signals. A signal processing pipeline specifically for developmental EEG hyperscanning studies was developed (Kayhan et al., 2022), and different ways to compute INS from EEG data were systematically compared (Marriott Haresign et al., 2022). The effects of different pre-processing strategies on parent-infant INS were tested (Pili et al., 2025) and practical guidelines for parent-infant EEG hyperscanning were developed (Turk et al., 2022).

**Fig. 2:**
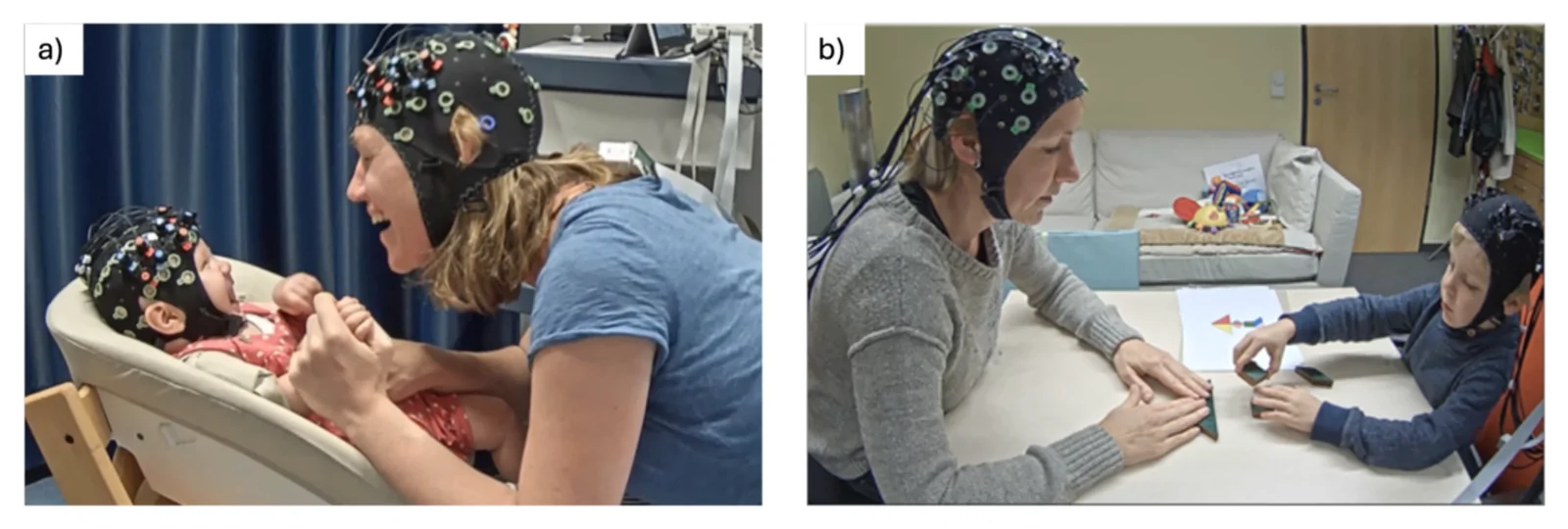
Example study setups for investigating INS in caregiver-infant (left, from Nguyen et al., 2023) and caregiver-child interactions (right, from Nguyen et al., 2020b).

Given the susceptibility of the EEG signal to facial, eye, and body movements, many researchers use functional near-infrared spectroscopy (fNIRS), which is less affected by motion artefacts, to assess INS during live social interactions. Unlike EEG, the focus is on the slower rhythms of hemodynamic activity in the brain. Most of the studies utilizing fNIRS have applied intersubject correlation (Piazza et al., 2020) or wavelet transform coherence (Reindl et al., 2018) as measures of INS. Nguyen and colleagues published an analysis pipeline and detailed guidelines on how to compute INS based on wavelet transform coherence from adult-child fNIRS hyperscanning data (Nguyen et al., 2021). Fig. 2 shows example study setups for studying INS in caregiver-infant and –child interactions.

Evidence for the spontaneous emergence of INS during social interactions between caregivers and their infants exists for infants as young as 4–6 months of age (Nguyen et al., 2021, 2023). Even at this young age, the level of caregiver-infant INS, assessed with fNIRS hyperscanning in frontal regions, is related to aspects of the interaction quality, including the duration of affectionate touch (Nguyen et al., 2021) and frequency of vocal turn-taking (Nguyen et al., 2023). It should be noted that INS reported in young infants and their caregivers might well be limited to slow rhythms captured by fNIRS, e.g., up to 0.3 Hz in the study by Nguyen et al. (2021), and lower frequency rhythms in the EEG, e.g., theta band activity between 4–7 Hz (Endevelt-Shapira et al., 2021). This is because the protracted process of postnatal myelination leads to relative increases in higher-frequency EEG activity with age as well as changes in the functional frequency bands of EEG oscillations (Saby & Marshall, 2012). In contrast, EEG hyperscanning studies with adults often also report alignment in higher frequency bands, such as gamma band activity up to 48 Hz (Dumas et al., 2010). Notably, high-frequency INS depends on intra-brain connections in the interacting partners (Moreau et al., 2022), which are still maturing across infancy (Gao et al., 2017). Furthermore, dominant EEG brain rhythms and boundaries of functional frequency bands are considerably lower in infants than in adults (Saby & Marshall, 2012), and communicative rhythms are often slowed down in interactions with infants (e.g., infant-directed speech). Yet, longitudinal research characterizing changes in INS across the first years and identifying implications for child development is currently lacking.

A systematic investigation of INS over developmental stages is warranted because, over time, the interactions between caregiver and child evolve and become more complex. Early social interactions in Western societies are typically dyadic in nature and become triadic during the first year of life (Striano & Reid, 2006). One early prerequisite for these interactions is mutual attention to each other (Reddy, 2003), complemented by intentional communication about objects of shared attention in the second half of the first year (Siposova & Carpenter, 2019). When involved in a social exchange, the caregiver and child need to visually track each other’s behaviors to respond adaptively and maintain the interaction. During the first year, caregiver-infant dyads tend to progressively occupy broader interaction spaces containing larger numbers of possible objects for play and, hence, increase the complexity of the social exchange (Schneider et al., 2023). One developmental hypothesis could thus be that INS temporarily decreases as caregiver-infant dyads progressively move towards broader interaction spaces. More specifically, INS may temporarily be reduced around major developmental milestones, such as the onset of infant locomotion (Campos et al., 2000), given the necessity to readjust mutual predictions and adaptations.

Yet, despite the growing complexity of caregiver-child social interactions, continuous exposure to each other’s behaviors generally allows the interaction to flow more smoothly over time. Mother-child interactions evolve between the first to the second year of life, with mothers typically showing an increase in sensitive behaviors and a decrease in intrusiveness (Perea-Velasco et al., 2023). This change in responsiveness is achieved through mutual engagement that is temporally coordinated and contingent in nature (Harrist & Waugh, 2002). In these synchronous interactions, one individual matches actions that are partly the partner’s and partly their own actions reflected back to them, with mutual prediction and adaptation being crucial in this process (Fogel, 2017). Mutual prediction and adaptation in caregiver-child interactions generally increase over time as both the caregiver and child become more attuned to each other’s behaviors, emotions, and needs (Hoehl & Bertenthal, 2021). Through repeated interactions, the caregiver learns to interpret and anticipate their infants’ signs of hunger, discomfort, or excitement, while the infant begins to recognize and respond to their caregiver’s patterns of speech, gestures, and emotional expressions. This growing behavioral synchronization fosters a sense of security and strengthens the bond between them, as well as facilitates the child’s ability to self-regulate over time (Bornstein & Esposito, 2023; Feldman, 2017; Stallworthy et al., 2024; Ulmer Yaniv et al., 2021). Early synchronous interactions help align children’s internal physiological and emotional experiences with their social partners and the environment, enabling them to interact more effortlessly with the world as they develop (Atzil et al., 2018; Feldman, 2007b; Gergely & Watson, 1996).

Mutual prediction is considered a key property of synchronous interactions, and INS may reflect the extent to which two individuals share mutual attention to common goals (Gvirts & Perlmutter, 2020), align their conceptual representations (Stolk et al., 2016), and generate more accurate predictions of their partner (Hamilton, 2021; Kingsbury et al., 2019; Koban et al., 2019; Mayo & Shamay-Tsoory, 2024). Many developmental studies investigating INS using fNIRS have focused on brain activity in the prefrontal cortex and TPJ, as these areas sustain mental state reasoning, self-other representation, and shared attention (Roche et al., 2025). These social brain regions undergo considerable structural and functional developmental changes in early childhood (Schneider et al., 2022) which are linked to both early attachment quality (Leblanc et al., 2017) and emerging social cognitive competences, such as theory of mind (Grosse Wiesmann et al., 2017). The predictive account of INS is further supported by experiments conducted in non-human animal models and human adults. For instance, through *in vivo* calcium imaging in interacting mice, Kingsbury et al. (2019) discovered that INS emerges as both social partners simultaneously monitor their own actions while predicting their partner’s behavior, leading to similar neural activity patterns. Building on this observation, Hamilton (2021) introduced an integrated framework that includes behavioral, physiological, and neural synchrony, emphasizing their role in embodied cognition and mutual prediction. Thus, despite the increasing complexity of caregiver-infant interactions across the first months, a developmental increase in INS is conceivable, especially in caregiver-child pairs who successfully co-create increasingly accurate predictive models of each other’s behavior. If confirmed in longitudinal research, INS may emerge as a useful biomarker for early interaction quality in addition to established behavioral coding schemes for caregiver-infant interactions that – unlike neural measures – may not capture subtle fluctuations in (mutual) attention and its alignment (Kulke, 2025; Wass et al., 2018).

### Developmental changes in leader–follower dynamics

So far, we have discussed how the degree of INS may vary across development in caregiver-child interactions. Yet, qualitative aspects of interpersonal brain dynamics, such as leader–follower dynamics, may also change across development. In adults, leader-–follower dynamics can arise due to social status asymmetry, situational requirements, or emerge spontaneously. In asymmetric social interactions, such as teaching scenarios, the amount of INS predicts the teaching outcome (Zhang et al., 2024), with the teacher’s brain activity often preceding the student’s (Pan et al., 2018, 2021; Zheng et al., 2018). Leader-follower dynamics also emerge spontaneously in adult dyads and groups in various tasks, from finger tapping to free conversations, even when the task *per se* does not require a clear distinction of roles (Jiang et al., 2015; Konvalinka et al., 2014). Leader-follower dynamics in adults have been linked to INS (Jiang et al., 2015; Konvalinka et al., 2014; Zhang et al., 2024) and to positive outcomes of social interactions, such as group bonding (Ni et al., 2024), behavioral alignment to leadership when under threat (Zhang et al., 2023) and creative problem-solving (He et al., 2023). Turn-taking and leader–follower patterns emerge as fundamental structures in both human and animal communication and have been argued to be evolutionarily conserved (Verga et al., 2023).

Given that social interactions between young children and their caregivers are naturally asymmetric, involving one more and one less mature partner, one might suspect that caregivers typically assume the role of the leader. Yet, decades of behavioral research into caregiver-infant interactions suggest otherwise, speaking to very early emerging reciprocal coordination with caregivers often following their infant’s lead (Beebe et al., 2010; Feldman, 2007b; Hilbrink et al., 2015; Jaffe et al., 2001). Social exchanges around 3 months of age are usually characterized by an infant-leads-caregiver-follows pattern, with time lags of about 2 s in the case of affect matching. This time lag decreases between 3 and 9 months, and the interactions become more bidirectional and mutually adaptive (Feldman et al., 1999). In vocal turn-taking, infant-caregiver latencies in this age range are even shorter, around one second, and the response latencies of infants and caregivers are correlated, suggesting mutual adjustment rather than unidirectional leader–follower patterns (T. Nguyen et al., 2023; V. Nguyen et al., 2022). Although infants start to actively direct their interaction partner’s attention through pointing by one year of age (Liszkowski et al., 2004), and are sensitive to their attention being followed (Grossmann et al., 2013; Phillips et al., 2023), joint attention episodes during interactive toy play remain largely driven by caregivers at this age (Phillips et al., 2023). Thus, both the temporal resolution and directionality of leader–follower dynamics depend on the mode of communication in caregiver-infant interactions. This makes the combination of hyperscanning with detailed behavioral analyses particularly informative.

Only a few studies have explored the temporal dynamics of INS in caregiver-infant dyads using methods such as Granger causality or time-lagged cross-correlations (Marriott Haresign et al., 2022). Using EEG hyperscanning during singing, Leong et al. (2017) found that mutual Granger-causal influences between an adult experimenter’s and 8-month-olds’ alpha and theta-band activities were stronger during direct eye contact compared to indirect gaze (Leong et al., 2017). They reported similar strengths of infant-to-adult and adult-to-infant influences at this age. Combining EEG hyperscanning with coding of caregivers’ and 12-month-old infants’ gaze during joint object play, Wass et al. (2018) revealed that parental theta power responded to infants’ gaze shifts and predicted longer periods of infant sustained attention, again stressing the existence of mutual influences between infants and adults (Wass et al., 2018).

Despite their lower temporal resolution, fNIRS hyperscanning studies are also informative regarding temporal dynamics of slower neural rhythms, especially when combined with behavioral coding. For instance, a study by Nguyen and colleagues showed that a higher frequency of caregiver-infant vocal turn-taking is related to medial prefrontal INS early on in a free play interaction at 4–6 months of age (Nguyen et al., 2023). Yet, this study did not analyze leader–follower dynamics in INS. In slightly older infants, at 9–15 months, Piazza and colleagues reported infant-adult INS during nursery rhyme singing, joint object play and picture book reading (Piazza et al., 2020). Using cross-correlations they found INS at zero lag and at lags of 1–3 s, with infant brain activity leading adult brain activity. Taken together with earlier behavioral findings, these results show that infants often assume leading roles in interactions with adults and that adults often adjust to infants. Yet, important questions remain open. Based on behavioral studies and research on infant brain maturation, changes in time lags can be expected, with INS likely peaking at shorter lags over time, given the gradual improvements in the temporal resolution of neural processing linked to changes in oscillatory brain activity throughout child development (Arioli et al., 2024; Freschl et al., 2022). Adapting behavior and brain activities to infants requires high levels of attention and sensitivity from their caregivers, which can be compromised by stress and psychopathologies, such as postpartum depression and anxiety (Feldman, 2007a). Accordingly, self-reported maternal stress has been linked to attenuated mother–child INS in several studies (Azhari et al., 2019; Nguyen et al., 2020b; St. Clair et al., 2025), with child irritability likely playing an aggravating role (Quiñones-Camacho et al., 2020). On the other hand, INS may be a protective factor against developing behavior problems in childhood (Quiñones-Camacho et al., 2022). Longitudinal research mapping out developmental changes in temporal dynamics, including leader–follower patterns of INS, is required to identify their role in both healthy development and psychopathology.

Furthermore, future research should characterize temporal dynamics in peer interactions across childhood and adolescence. Compared to caregiver-child interactions, measuring INS in child-peer interactions remains a significant research gap. However, existing behavioral research offers insights that can guide future studies including neural measures. For example, Stivers and colleagues examined how children aged 4–8 years take turns in question-response interactions during peer conversations (Stivers et al., 2018). Their findings show that children’s timing and response patterns gradually become more structured. However, children do not yet fully recognize when their responses follow or break social rules, leading to more frequent delays or missing responses in conversation (Stivers et al., 2018). Another study found that timing in coordinated actions strengthens social bonds in five-year-old peers (Wan & Zhu, 2021). Specifically, precise and frequent rhythmic coordination, where children alternated playing percussion instruments every beat, led to stronger social effects than less frequent structured turns. This structured rhythmical interaction mirrors the predictability and mutual adaptation seen in turn-taking, which is fundamental to social interactions (Wan & Zhu, 2021). Based on these findings from behavioral research, increasing levels of INS may be expected in child-peer dyads with growing temporal coordination skills and stronger social bonds.

### The role of language and cognitive development in INS

Above and beyond rhythmic coordination, language acquisition and higher-order cognitive development likely impact the degree of INS that children achieve in interactions with adults and with peers. This should especially be the case in social interactions that require common ground in the sense of shared semantic representations and understanding. Research in adults has shown that INS is modulated by factors such as co-presence and prior social engagement (De Felice et al., 2024), with similarity in narrative interpretation playing a crucial role (Nguyen et al., 2019). Friends, in particular, tend to exhibit more similar neural processing of narratives (Parkinson et al., 2018). Simply put, INS in situations requiring higher-order semantic processing is increased between individuals who understand and interpret the world more similarly. Given these insights from research with adults, examining how the developmental trajectories of language acquisition, perspective-taking, and social conventions shape the emergence and quality of INS in childhood is essential.

A fundamental driver of INS should be language development, which enables children to establish common ground with others. Language acquisition involves mastering syntax, semantics, and pragmatics, i.e., the ability to use language effectively in social contexts. These foundational skills develop through rich social interactions, where children learn to interpret and predict others’ intentions and receive contingent feedback in response to their own vocalizations (Elmlinger et al., 2019; Goldstein et al., 2003; Tamis-LeMonda et al., 2001). Through conversational turn-taking, dialogue-based perspective-taking, and the implicit rules of communication, children enhance the predictability and alignment of social interactions (Clark, 1996). Even in infancy, joint attention and synchronized activities, such as shared book reading, provide a foundation for semantic learning and mutual understanding (Piazza et al., 2021). During shared book reading, for example, caregivers label objects, describe actions, and engage infants in turn-taking, all of which help build early word associations and reinforce shared understanding. As interactions become more complex, children expand their vocabulary and refine their conceptual understanding to fully integrate and coordinate these shared representations with their own perspectives. During the preschool years, children’s linguistic abilities expand rapidly. They acquire a broader lexicon (Goodman, 1997) and gain a deeper understanding of syntactic structures, enabling more complex and nuanced communication (Tomasello, 2003). These advances are closely linked to brain maturation, especially functional connectivity between language processing brain areas (Friederici et al., 2017), and likely support the emergence of INS in narrative-based and collaborative activities that require joint interpretation and reasoning (Dikker et al., 2017; Nguyen et al., 2020a). As children’s language skills develop, they also refine their ability to navigate social interactions through pragmatic cues, such as tone of voice, conversational implicatures, and cultural norms of dialogue (Gelman & Raman, 2003; Groba et al., 2018).

In addition to language abilities, social cognitive development likely affects INS in the early years. Whereas children are able to represent the goals, intentions, and wishes of other people in the first 1.5 years (Meltzoff, 1995; Repacholi & Gopnik, 1997; Woodward, 1998), complex mentalizing abilities undergo a protracted development. Explicit understanding of other people’s true and false beliefs is achieved by around 4 years of age, related to white matter maturation in TPJ (Grosse Wiesmann et al., 2017; Wellman et al., 2001), enabling children to mentally represent and verbally communicate about others’ mental states. Furthermore, children acquire the social norms and conventions of their cultural environment from around 3 years of age (Rakoczy et al., 2008). Sharing a general understanding of how one ought to behave is likely conducive to building social connections (Delgado et al., 2023) and increases mutual predictability (Köster et al., 2020; Veissiere et al., 2019).

Studies using intersubject correlation to operationalize INS driven by shared processing of higher-order semantic information, such as movies and narrated stories, provide valuable insights into how these abilities develop. For example, a study on naturalistic movie viewing showed that children aged 4 to 6 years exhibit more variable brain activation patterns compared to adults, with intersubject correlation increasing among peers as they age (Moraczewski et al., 2018). This could reflect increasing functional specialization when processing socially meaningful stimuli. At the same time, it could also reflect increasingly shared understanding of narratives between children as they become socialized in their cultural environment (Piipponen & Karlsson, 2021).

Taken together we hypothesize that INS in tasks requiring higher-order understanding of abstract concepts, mental states, or social norms will increase with advanced mentalizing skills and socialization experiences and maturation of language and social brain areas across the preschool age, although longitudinal evidence from hyperscanning research is currently missing. This likely applies to children’s synchronization with adults and peers as they become immersed in their cultural environments. An open question concerns socialization processes and development beyond the preschool years. In adolescence, increased orientation towards peers (Laursen & Veenstra, 2021; Lew-Levy et al., 2023) may result in higher levels of INS among peer dyads compared to adults (for initial evidence, see Yang et al., 2023). Research also points towards a direct relationship between INS and social competence in adolescence, as initial evidence from autistic and neurotypical adolescents suggests (Key et al., 2022). For future research, it will thus be important to extend efforts beyond early childhood to get a fuller picture of developmental processes shaping INS and social connection across different phases of human ontogeny.

## Interpersonal neural synchrony as a developmental mechanism?

**Fig. 3:**
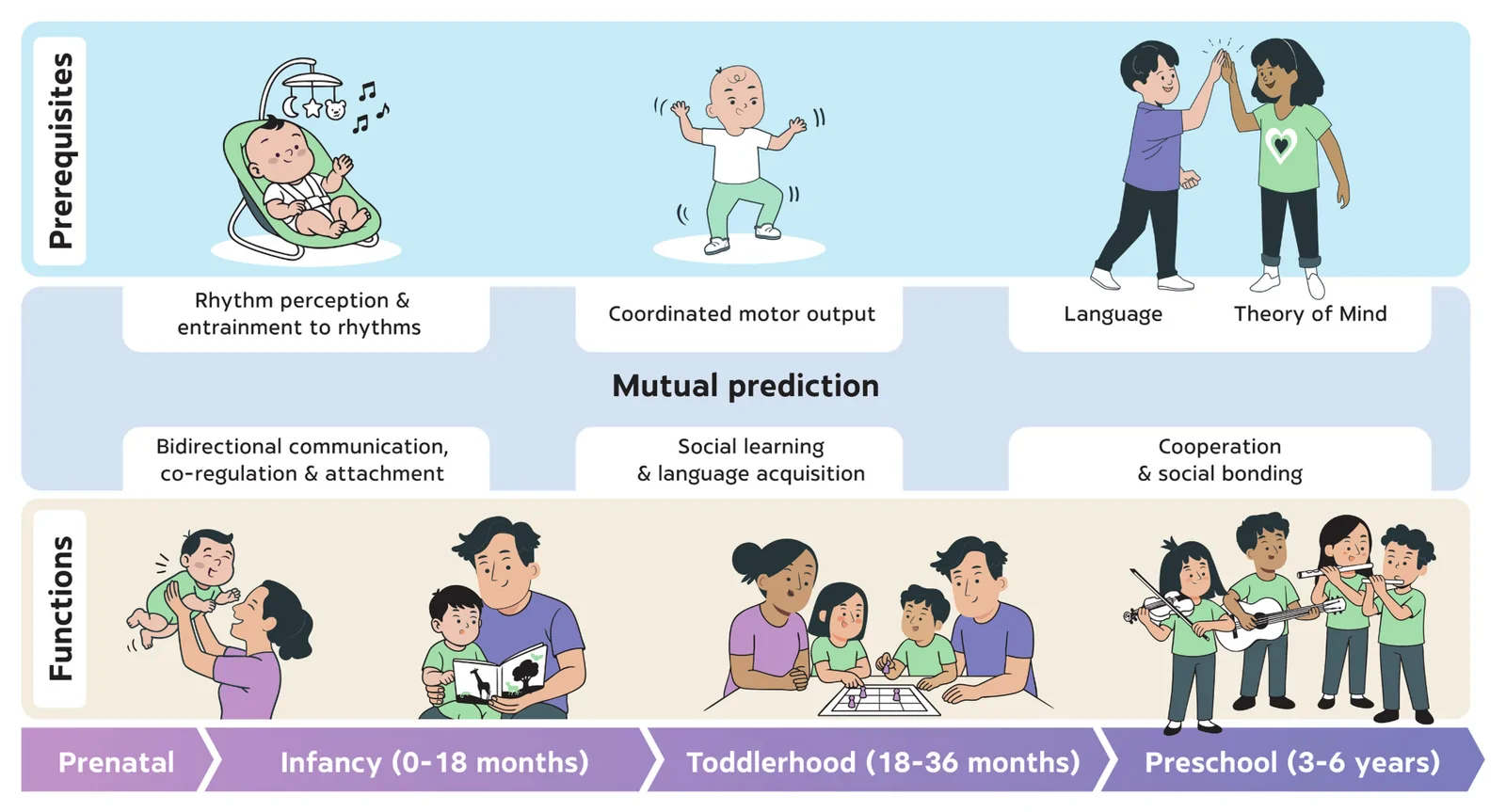
Overview of the developmental prerequisites and proposed functions of INS in early child development. Image by Adi Ramot.

In the previous sections, we have outlined the development of neural, cognitive, and social processes associated with the emergence of INS in social interactions (see also Fig. 3). Given the scope and complexity of the described developmental changes, it is important to consider their developmental trajectories in INS research, both for designing hyperscanning experiments with developmental populations and interpreting their results. We will now turn to the question of whether and how the presence of INS itself may have an impact on child development. Is INS in early caregiver-child interactions conducive to child development? May it even present a developmental mechanism (Benton, 2023), which underlies developmental change and could, in principle, be manipulated to improve developmental outcomes? Given the dearth of longitudinal research in this field, this important question is far from comprehensively answered (Roche et al., 2025). In the following section, we therefore point to promising theoretical considerations and potential avenues for future research, including research on social learning, language acquisition, and rhythm-based interventions.

### Early social learning and language acquisition

INS in teacher-learner interactions has been consistently related to learning outcomes in adults (Kostorz et al., 2020; Pan et al., 2018, 2021; Zhang et al., 2022; Zhang et al., 2024). Complementing correlational evidence, results from multibrain stimulation studies even point to causal relations between INS and social learning outcomes (Novembre & Iannetti, 2021). In these studies, brain activities of multiple participants are stimulated, e.g. through non-invasive neuromodulation methods such as transcranial alternating current stimulation. Thus, INS can be experimentally induced and effects on behavior and cognition can be tested. In one notable study, synchronized transcranial alternating current stimulation of the inferior frontal brain regions of learner and instructor during teaching resulted in synchronized movement and, importantly, enhanced performance in a song-learning task (Pan et al., 2020). This finding speaks to a potential causal role of INS in promoting information transfer from one person to another. According to the *interbrain-plasticity account*, interaction-based learning, such as song learning, induces changes in short- and long-term patterns of INS between teacher and learner, facilitating information transfer over time (Shamay-Tsoory, 2021). Following this proposition, children’s and their caregivers’ and teachers’ brains may, over repeated learning interactions, establish increasingly intricate inter-brain connectivity patterns. These connectivity patterns may promote the establishment of INS over time, supporting social learning and the co-creation of shared representations.

Future longitudinal research is required to directly test the hypothesis of INS promoting information transfer in early development, but preliminary evidence suggests that this is a promising research avenue. For instance, 9-month-old infants’ brains entrain especially well to the prosodic rhythm of their mothers’ infant-directed compared to adult-directed speech (Menn et al., 2022). In this study, mothers spontaneously modulated prosodic stress amplitudes when engaging in infant-directed speech. Thus, mothers seem to intuitively modulate aspects of their speech to facilitate their infants’ neural synchronization to their voice. Although not directly tested in this study, this may foster infants’ language acquisition. Indeed, another study showed that 7-month-olds’ neural and motor tracking of their caregivers’ infant-directed singing predicts their vocabulary size at 20 months (Nguyen et al., 2023). Recent evidence also suggests that newborns’ cortical speech tracking relates to their language development at 6 months of age (Florea et al., 2024).

Caregivers adapt their speech (Menn et al., 2022), use gaze (Abney et al., 2020), refer to infants by name (Parise et al., 2010), and modulate their movements during demonstrations (Van Schaik et al., 2020) to support infants’ understanding of the world. These multimodal cues may facilitate early learning by modulating infants’ neural oscillatory dynamics, particularly in the theta band, a rhythm associated with infant learning (Begus & Bonawitz, 2020). Neural entrainment to communicative signals in the theta-band may stimulate infants’ theta brain activity, potentially enhancing memory encoding and access to semantic systems (Hoehl et al., 2014; Michel et al., 2024). Michel and colleagues (2024) investigated whether maternal communicative behaviors influence infants’ thetaband EEG activity during object encoding, comparing adult-directed speech with an infant-directed condition that additionally included gaze and name-calling. Although theta power did not differ significantly between conditions, both types of engagement elicited greater theta activity than a non-social resting phase, and theta power during the encoding phase predicted infants’ later object recognition. These findings suggest that communicative signals can modulate infants’ neural dynamics, with particular importance for oscillatory bands implicated in early learning. Future research should further explore how different types of communicative input influence theta-band activity and support learning, potentially linking neural processing and INS during encoding with later memory retrieval processes.

Corroborating evidence for a potential role of INS in children’s social learning and language acquisition comes from research with 3–4-year-old children (Piazza et al., 2021). In this study, children engaged in joint book reading with an adult experimenter. Novel words and objects were embedded in the story. INS in the parietal cortex between children and the experimenter during joint book reading was positively related to their learning of novel words from the book. This research is important as it demonstrates how INS is spontaneously established in naturalistic learning interactions between children and adults (Besser Ilan et al., 2024). A study with high school students further demonstrated the feasibility of assessing links between INS and learning in real-world educational contexts (Dikker et al., 2017). Yet, correlational studies cannot unambiguously answer whether INS is a mechanism or a correlate of information sharing and transfer between the adult and child brain. Experimental evidence, ideally through non-invasive manipulation of INS during learning interactions, as done in studies with adults (Pan et al., 2020), would be necessary to substantiate stronger claims regarding the mechanistic role of INS for social learning in infancy and childhood. Apart from multibrain stimulation that comes with some ethical concerns in developmental research, perceptual rhythmic stimulation (Köster et al., 2023) or rhythm-based interventions, as discussed next, might present promising avenues to test mechanistic hypotheses on the role of INS for children’s social learning.

### Rhythm-based interventions

Rhythm-based interventions, particularly those including musical elements, provide a powerful means of fostering social coordination and bonding through entrainment (Gerry et al., 2012). These interventions, conducted in interactive contexts, incorporate shared rhythmic experiences that are likely to enhance interpersonal synchrony, including INS. Although rhythm processing is often a primary target, music interventions presumably operate through multiple pathways. These include reward systems, where the pleasurable aspects of music listening and creation may drive engagement; arousal regulation, which can stabilize or elevate mood depending on the context; and self-regulation, whereby rhythmic patterns may help scaffold attention or behavior (Fiveash et al., 2023).

Rhythm-based interventions have shown promise in diverse populations. In children, rhythm perception is crucial for language processing, and deficits in neural entrainment to speech rhythms have been linked to dyslexia (Goswami, 2011). Interventions such as drumming (Thomson et al., 2013), rhythm and pitch training (Patscheke et al., 2019), or moving to a beat (Frey et al., 2022) improve rhythm discrimination and synchronization, suggesting broader benefits beyond literacy.

Similarly, during pregnancy (Corbijn Van Willenswaard et al., 2017), activities such as listening to music (Baltacı et al., 2024; Chang et al., 2008; Yang et al., 2009), singing (Wulff et al., 2021), and dancing (Arioli et al., 2025; Branson Dame et al., 2024) were suggested as a means to reduce the negative effects of stress. While some of the interventions centered on parental wellbeing (Baltacı et al., 2024; Bauer et al., 2010; Chang et al., 2008), others aimed at processes within the parent-infant dyad, including emotional connection (Branson Dame et al., 2024) and bonding (Persico et al., 2017; Wulff et al., 2021).

Studies on preterm infants highlight how maternal singing and music exposure can enhance social engagement, emotional regulation, and early parent-infant bonding (Arnon et al., 2006; Filippa et al., 2013; Lejeune et al., 2019; Malloch et al., 2012). Similarly, in autistic children, musical activities have been shown to improve social responsiveness, likely by strengthening mechanisms of auditory-motor entrainment (Lense & Camarata, 2020; Sharda et al., 2019). More specifically, a study has shown that dyadic drum playing increased the interpersonal coordination skills of autistic children (Yoo & Kim, 2018).

In sum, a number of existing rhythm-based interventions implemented across a variety of contexts and developmental populations likely have implications for INS, either as a possible consequence of general rhythm-processing improvement (e.g. interventions in the context of literacy development), or as a putative mechanism through which these interventions operate (e.g. interventions with autistic children). Future research should examine directly whether an enhancement of INS can be observed as an effect of rhythm-based interventions, and whether this enhancement relates to behavioral outcomes. Finally, interventions explicitly targeting INS as an outcome, for instance, through hyperscanning neurofeedback (Kostorz et al., 2025; Dikker et al., 2019), are a promising avenue for research on supporting dyadic interactions across development.

### Attachment formation and emotion regulation

In addition to its putative role in social learning and language acquisition, INS has been proposed as a mechanism underlying attachment formation and emotion regulation in childhood (Feldman, 2007b; Reindl et al., 2018). According to the *biobehavioral synchrony model*, early experiences of behavioral, physiological, and neural synchrony in caregiver-infant interactions shape the formation of attachment bonds across the lifespan (Feldman, 2017). In this view, experiences of synchrony, including INS, early in life, influence social brain development, promote children’s self-regulation, and predict the quality of their later attachment relationships. Sensitive caregivers may establish INS with their infants, for instance, through affectionate touch (Nguyen et al., 2021), to regulate arousal levels and signal the availability of reliable and predictable caregiving (Atzil et al., 2018). Indeed, a range of hyperscanning studies have documented a link between the degree of closeness in personal relationships and INS levels in both adults and children (De Felice et al., 2025; Gvirts & Perlmutter, 2020; Wheatley et al., 2012).

One notable study showed that cooperation (compared to competition) leads to increased levels of frontal brain INS, specifically between 5–9-year-old children and their caregivers, but not adult strangers (Reindl et al., 2018). Importantly, higher INS with the parent compared to a stranger was associated with better emotional regulation in both parents and children. Results of a mediation model suggested that parental reappraisal, as an emotion regulation strategy, was linked to higher levels of INS during parent–child cooperation, which may positively affect the child’s emotion regulation abilities. The authors argue that INS may represent a mechanism supporting the formation of early bonds and a pathway through which parental emotion regulation influences the child’s emotional development, in addition to genetic links and social learning. A recent study applying EEG hyperscanning also found that 6-month-old infants show enhanced INS when interacting with their mothers compared to strangers (Endevelt-Shapira et al., 2021). Interestingly, this difference was attenuated in the presence of maternal odor, indicating that maternal chemosignals may facilitate alloparenting in early human development.

Based on these cross-sectional findings, longitudinal research is needed to further clarify the functional role of INS for early attachment formation. An impressive longitudinal study has recently linked the neonatal experience of mother-infant body contact to behaviorally synchronous interactions across childhood and the development of brain regions implied in emotion regulation and empathy twenty years later (Ulmer Yaniv et al., 2021). While these results point to (behavioral) synchrony as a potential mechanism underlying social brain development, it should be noted that caregiver-infant interactions probably benefit most from high, but not extreme, levels of interactional contingency and synchrony (Beebe et al., 2010). According to Beebe’s *optimal mid-range model*, both too high and too low levels of maternal behavioral contingency are indicative of inadequate sensitivity and are associated with insecure attachment. Interestingly, while the positive effects of (induced) behavioral synchrony on affiliation and prosocial behavior are well-documented in adults (Hove & Risen, 2009) and infants (Cirelli et al., 2014), recent research has shown that adults prefer a combination of synchrony and complexity in spontaneous motor synchrony tasks (Ravreby et al., 2022).

A balance between synchrony and maintaining interest through an appropriate amount of complexity might be more effective than maximizing synchrony for establishing positive social bonds. The same might be true in infancy, where exceeding levels of synchrony can be intrusive (Beebe et al., 2010; Smith et al., 2022). Alternatively, adaptive levels of mutual predictability and synchrony might vary across interactional contexts and developmental stages. Research quantifying the predictability of infant-directed speech has shown that parents use more repetitive and redundant speech with younger infants and reduce the predictability of their speech as children grow (Tal et al., 2024). This age-related change in complexity could optimally stimulate children’s early language learning, adapted to their respective developmental stage. Similarly, early caregiver-infant interactions might benefit from higher levels of predictability and synchrony (Vanoncini et al., 2022), whereas more complexity and flexibility might be beneficial later on (Gordon et al., 2025).

Unfortunately, hyperscanning research directly linking INS with caregiver-child attachment remains scarce. A series of recent studies showed that 5-year-olds establish INS with their mothers (Nguyen et al., 2020a,b) and fathers (Nguyen et al., 2021) during collaborative problem-solving. Whereas higher levels of INS in mother–child pairs were associated with higher levels of behavioral reciprocity, the same patterns were not found in father-child pairs. INS in father-child dyads was related to the extent to which fathers identified with their role as a parent. Thus, fathers and mothers may establish INS through different behavioral patterns (Feldman et al., 2019), stressing the importance of extending research on INS and early caregiving to all caregivers, including mothers and fathers. A large hyperscanning study including mothers and fathers and their preschool-aged children also reported gender-specific relations between INS and parental attachment representations (Nguyen et al., 2024). Specifically, increased levels of INS were observed in mother–child pairs in which mothers reported insecure attachment representations based on their own attachment history. This may point to compensatory efforts on the part of these mothers. Whether and how these patterns influence the quality of the parent–child relationship and INS in the long run remains a topic of future research.

## Future directions

Throughout our paper, we have pointed to extant knowledge gaps relating to INS in early human development. A key challenge for future research adopting a developmental perspective on INS is to further explore its role in both typical and atypical developmental trajectories. In addition to studying group-level effects, the consequences of matches and mismatches in personality traits within dyads might be of great interest, given that social experiences both shape and are shaped by psychological factors such as personality traits. For instance, future studies could examine how children’s temperament shapes the quality of their social interactions and INS with caregivers in early life and, subsequently, with peers. In particular, the goodness of fit of a child’s temperament with environmental demands and caregivers’ personality traits may play a role (Thomas & Chess, 1977). Preliminary evidence showed a role of personality traits in predicting INS across experimental tasks in adolescents and adults (Dikker et al., 2017; Lim et al., 2024; Zhang et al., 2021). However, a study by Nguyen et al. observed that state-like factors (e.g., maternal stress) may play a more prominent role in predicting INS than trait-like factors (e.g., child temperament) in mother–child interactions (Nguyen et al., 2020b). Investigating these associations longitudinally could provide valuable insights into how early dispositional and psychological traits contribute to the development of INS and social competencies over time.

Moreover, social experiences and social-cognitive skills are central to many atypical developmental trajectories. For example, autism is characterized by persistent challenges in social communication and interaction. A growing number of hyperscanning studies have now indicated that INS is decreased in dyads consisting of autistic and neurotypical individuals (Hirsch et al., 2022; Tanabe et al., 2012). In such dyads, social competence positively correlates with INS (Key et al., 2022). Similar results were observed in interactions of autistic children and their caregivers during a cooperative task (Wang et al., 2020). Specifically, the authors observed that the severity of autism symptoms in children negatively relates to INS when interacting with their parents. However, stemming from the observation that social challenges seem to be less pronounced when two autistic people interact with each other, recent hyperscanning work compared INS between dyads that were either matched or mismatched in autistic-like traits (Bolis et al., 2017; Kruppa et al., 2021; Minagawa et al., 2023; Moreau et al., 2024; Schilbach & Redcay, 2025). Peng et al. (2024) found that while dyads of individuals with high autistic traits exhibited distinct communicative behaviors compared to other groups, their INS was actually greater (Peng et al., 2024). This finding highlights the need for further research to systematically examine how interpersonal differences influence social interactions and their neural correlates.

Similarly, disruptions in social connectedness are a core feature of various mental health conditions (Schilbach & Redcay, 2025).

For instance, individuals with depression often experience a profound sense of social disconnection. In contrast, those with anxiety, particularly social anxiety, may struggle with overly synchronizing their behavior and emotions with others in social interactions. Examining INS in these populations could provide new perspectives on how neural synchrony contributes to social difficulties and whether interventions aimed at modulating synchrony could support social functioning and mental well-being (Konrad et al., 2024). From a developmental perspective, it will be of special interest to investigate how caregiver-child INS relates to both caregiver and child mental health, in the current moment as well as longitudinally, and how INS can contribute to resilient functioning (Konrad & Puetz, 2024). Along these lines, a recent study by Zhang and colleagues found reduced mother–child synchrony in children with oppositional defiant disorder (Zhang et al., 2024), but corresponding research remains sparse. This is of interest, as early attachment is related to child and adolescent mental health (Cassidy et al., 2013; Warren et al., 1997), and attachment is thought to relate to INS (Feldman, 2017; Nguyen et al., 2024).

A grave limitation of existing work pertains to the lack of longitudinal research linking INS with developmental variables and outcomes over time. Strong claims for INS as a developmental mechanism should rely on such empirical links and the potential to modulate INS – and thus improve the associated developmental outcomes – in caregiver-child interactions. Besides being costly, such research efforts present considerable conceptual and methodological challenges. Cross-generational hyperscanning research has to deal with age-related differences and changes in the frequency spectrum of bodily and brain rhythms (Klimesch, 2013). Systematic approaches to tackle the interplay of INS and age-related changes in neurophysiological processes over the lifespan have only recently been proposed (Dikker et al., 2024). Given that endogenous rhythms (Zamm et al., 2016) and intrapersonal neural connectivity (Moreau et al., 2022) impact interpersonal synchronization – and underlie substantive developmental changes in early childhood – it is of great importance to systematically map out the links between neurophysiological changes and INS within and across different age groups, from young infants to older adults. Complementing common measures of INS that usually assess synchrony in the same frequency band across brains, cross-frequency synchrony measures might add additional insights (Kayhan et al., 2022). Studying INS in a developmental context thus remains key to understanding typical and atypical social functioning.

## Conclusion

Infants are sensitive to internal and external perceptual rhythms from early on in life. Yet, their ability to synchronize with communicative rhythms undergoes considerable development. Processes of brain maturation support increasing temporal precision, sensitivity for complex social rhythms, and the ability to produce synchronized motor responses within the first years. Despite initial limitations in temporal precision, INS emerges spontaneously in caregiver-infant interactions as early as four months of age. As longitudinal research into the development of INS is currently lacking, we have proposed a set of developmental hypotheses based on behavioral and neuroscience research to be tested in future studies. Specifically, we propose that INS may initially be constrained to low-frequency brain rhythms and that both the degree of INS and qualitative leader–follower patterns likely change across early development, along with variations in mutual predictability between children and caregivers. We further hypothesize that INS during tasks requiring higher-order mutual understanding is linked to progress in language acquisition and socio-cognitive development, allowing children to establish common ground with their caregivers and peers. Longitudinal research, potentially implementing rhythm-based interventions, is required to corroborate the potential role of INS as a developmental mechanism underlying social learning and attachment formation. This research will have great potential to deepen our understanding of child development, and it could point to new pathways for supporting social learning and attachment in caregivers and children with elevated risks for psychopathology and impaired social bonding.

## Data Availability

No data was used for the research described in the article.
